# Fine-Tuning the Wall Thickness of Ordered Mesoporous Graphene by Exploiting Ligand Exchange of Colloidal Nanocrystals

**DOI:** 10.3389/fchem.2017.00117

**Published:** 2017-12-13

**Authors:** Dandan Han, Yancui Yan, Jishi Wei, Biwei Wang, Tongtao Li, Guannan Guo, Dong Yang, Songhai Xie, Angang Dong

**Affiliations:** ^1^Shanghai Key Laboratory of Molecular Catalysis and Innovative Materials, Department of Chemistry, Collaborative Innovation Center of Chemistry for Energy Materials, Fudan University, Shanghai, China; ^2^Department of Macromolecular Science, Fudan University, Shanghai, China

**Keywords:** ordered mesoporous graphene, graphene layer thickness, ligand exchange, self-assembly, colloidal nanocrystals

## Abstract

Because of their unique physical properties, three-dimensional (3D) graphene has attracted enormous attention over the past years. However, it is still a challenge to precisely control the layer thickness of 3D graphene. Here, we report a novel strategy to rationally adjust the wall thickness of ordered mesoporous graphene (OMG). By taking advantage of ligand exchange capability of colloidal Fe_3_O_4_ nanocrystals, we are able to fine-tune the wall thickness of OMG from 2 to 6 layers of graphene. When evaluated as electrocatalyst for oxygen reduction reaction upon S and N doping, the 4-layer OMG is found to show better catalytic performance compared with their 2- and 6-layer counterparts, which we attribute to the enhanced exposure of active sites arising from the thin wall thickness and high surface area.

## Introduction

Owing to the superior electrical conductivity, high specific surface area, and excellent mechanical and electrochemical stability, grapheme, and few-layer graphene have been considered as promising materials for applications in electronics, sensing, catalysis, and energy storage (Geim and Novoselov, 2007; Geim, 2009; Hu et al., 2013; Han et al., 2014). In particular, three-dimensional (3D) frameworks constructed from stacked graphene layers are emerging as a new class of graphene-based materials, which have attracted increasingly greater attention because of their interconnected porous structures and abundant porosity plus high electrical conductivity inherited from 2D graphene (Choi et al., 2012; Xu et al., 2015; Yu et al., 2015). In addition to the porosity such as pore size and pore ordering, the wall thickness, i.e., the number of graphene layers (Kim et al., 2003; Luo et al., 2009; Huang et al., 2012), is another important parameter that cannot be ignored, as it dictates the accessibility of active sites, interlayer resistance, and structural stability of 3D graphene (Ferralis et al., 2008; Liu et al., 2008; Cho et al., 2011; Li et al., 2013). All these characteristics strongly influence the application and performance of 3D graphene. Previous methods to construct 3D graphene typically rely on the self-assembly graphene oxide sheets (Jiang et al., 2010) or template-directed growth (Yang et al., 2016). Although 3D graphene is obtainable by these methods, it is difficult to control the porosity and the graphene layer thickness simultaneously.

Current methods of adjusting the layer number of graphene involve the chemical vapor deposition (CVD) on transition metals such as Ni and Cu (Reina et al., 2009; Yan et al., 2011). For instance, Chen et al. reported the fabrication of macroscopic graphene frameworks by template-directed CVD growth, where one can control the average number of graphene layers by changing the precursor concentration (Chen et al., 2011). Despite the possibility to tailor the layer thickness by these CVD-based methods, the resulting 3D graphene usually possesses a disordered porous structure with poorly controlled pore sizes (Wu et al., 2012; Zhao et al., 2012). To address these issues, our group has recently developed a bottom-up strategy, based on the conversion of self-assembled nanocrystal (NC) superlattices, enabling the growth of ordered mesoporous graphene (OMG) with a high degree of graphitization and high surface areas (Jiao et al., 2015). The resulting OMG, derived from the native hydrocarbon ligands such as oleic acid (OA) attached to the NC surface, is constructed by few-layer graphene, possessing a highly ordered mesoporous structure inherited from the close-packed structure of NC superlattices (Dong, 2015). We note that although the pore size and shape of OMG can be readily controlled by tuning those of NC building blocks, it is still a big challenge to fine-tune the wall thickness of OMG.

Herein, we report a novel strategy to rationally adjust the number of graphene layers without compromising the structural ordering of OMG by exploiting the ligand exchange capability of colloidal NCs. We show that by adjusting the chain length of the ligands tethered to the Fe_3_O_4_ NC surface through ligand exchange, we are able to tailor the carbon content and therefore the average number of graphene layers in the resulting OMG. To demonstrate the importance of tuning wall thickness, the resulting OMG having different wall thicknesses, upon doping with N and S, are employed as electrocatalysts for oxygen reduction reaction (ORR). It is found that the 4-layer OMG exhibits a prominent catalytic activity with a half-wave potential at 0.80 V, which is lower than that of their 2- and 6-layer counterparts.

## Experimental

### Materials

Oleic acid (OA, 90%), oleylamine (OAm, 90%), 1-octadecene (ODE, 90%), octylamine (90%), butylamine (99%), thiourea (99.0%), and Nafion (5 wt%, contains 15-20% water) were purchased from Aldrich. Terafluoboric acid (HBF_4_, aqueous solution, >40%), N, N-dimethylformamide (DMF), and hexane were purchased from Aladdin. Sodium oleate and iron chloride hexahydrate (FeCl_3_.6H_2_O) were obtained from TCI. Pt/C (20 wt% of Pt) catalyst was purchased from Johnson Matthey. All chemicals were used as received without further purification.

### Synthesis of Fe_3_O_4_ NCs

Monodisperse 14 nm Fe_3_O_4_ NCs were synthesized and purified based on the literature method (Park et al., 2004). The as-synthesized, OA-capped Fe_3_O_4_ NCs are dispersed in hexane to form stable colloidal solutions with a concentration of about 10 mg mL^−1^.

### Ligand-exchange reactions of Fe_3_O_4_ NCs

A two-step ligand-exchange process is employed to treat the original OA-capped Fe_3_O_4_ NCs, resulting in various surface-coating hydrocarbon moieties. The first ligand-exchange reaction involves the use of HBF_4_ as the surface-treatment agent. In a typical procedure, 10 mL of Fe_3_O_4_ NC solution in hexane was mixed with 10 mL of DMF at room temperature to form a two-phase mixture, into which an appropriate amount of HBF_4_ solution was added dropwise. The resulting mixture was vigorously stirred until Fe_3_O_4_ NCs were completely transferred from hexane to the bottom DMF phase. To remove the excess HBF_4_, 15 mL of toluene was added to flocculate Fe_3_O_4_ NCs, which were collected by centrifugation. The ${\text{BF}}_{4}^{-}$-capped Fe_3_O_4_ NCs were dispersed in DMF to form stable colloidal dispersions. The second ligand-exchange procedure was carried out similarly using various hydrocarbon ligands such as butylamine, octylamine, or octylamine/OAm as the surface-treatment agent, transferring Fe_3_O_4_ NCs back to the hexane phase. After purification, the resulting hydrocarbon-capped Fe_3_O_4_ NCs were dispersed in hexane to form stable colloidal solution with a concentration of about 10 mg mL^−1^ for subsequent self-assembly.

### Synthesis of OMG

3D superlattices consisting of close-packed Fe_3_O_4_ NCs capped by different hydrocarbon ligands (e.g., octylamine, octylamine/OAm, and OA, etc.) were all obtained by a solvent-evaporation-based assembly method under ambient conditions. The as-assembled Fe_3_O_4_ superlattices were calcined at 500°C in Ar for 2 h and then treated by HCl to afford mesoporous carbon frameworks. The amorphous carbon frameworks obtained were then calcined at 1,000°C in Ar for 2 h, resulting in OMG with different wall thicknesses depending on the surface-coating hydrocarbon ligands.

### Synthesis of N, S Co-doped OMG

Thiourea was employed as the N and S sources for doping the graphene frameworks. In a typical procedure, 10 mg of OMG was mixed with 100 mg of thiourea in a porcelain boat, which was then heated in a quartz tube furnace at 900°C under Ar for 1 h.

### Characterization

Transmission electron microscopy (TEM), scanning TEM (STEM), high-resolution TEM (HRTEM), and energy-dispersive X-ray spectroscopy (EDS) and elemental mapping were performed on a Tecnai G2 20 TWIN microscope operated at 200 kV. Field-emission scanning electron microscopy (FESEM) images were collected on a Zeiss Ultra-55 microscope operated at 5 kV. Small-angle X-ray scattering (SAXS) was performed on a Nanostar U small angle X-ray scattering system using Cu Ka radiation (40 kV, 35 mA). Powder X-ray diffraction (XRD) was conducted on a Bruker D4 X-ray diffractometer. Fourier-transform infrared (FTIR) spectra were acquired using a PerkinElmer Spectrum Two spectrometer with a scanning range from 4,000 to 400 cm^−1^. Nitrogen adsorption–desorption isotherms were recorded on a Tristar 3000 instrument. Raman spectra were measured on an XploRA Raman system at room temperature. Thermogravimetric analysis (TGA) measurements were carried out on a Perkin–Elmer Pyris 1 thermogravimetric analyzer.

### Electrochemical measurements

All electrochemical measurements were performed in a standard three-electrode cell using an electrochemical station (CHI 660D), with Pt/C and saturated calomel electrode serving as counter and reference electrodes, respectively. All half-wave potentials reported in this work were converted into potentials against the reversible hydrogen electrode (RHE). In the preparation of the working electrode, catalyst (1 mg) and 6 μL Nafion solution (5 wt%) were dispersed in 250 mL of ethanol ultrasonically until a homogeneous ink was formed. Then, 6 μL of the catalyst ink was pipetted onto glassy carbon electrodes and dried naturally for further electrochemical tests. Before test, pure N_2_ or O_2_ was bubbled into 0.1 M KOH electrolyte for 30 min. Cyclic voltammetry (CV) was carried out from 0.2 to 1.2 V with a scan rate of 50 mV s^−1^. Linear sweep voltammetry (LSV) polarization curves were measured with a sweep rate of 10 mV s^−1^ at a rotating speed of 1,600 rpm.

## Result and discussion

### Fabrication procedure

As the OMG is derived from the hydrocarbon ligands stabilizing Fe_3_O_4_ NCs, one can reasonably expect that tuning the ligand chain length and/or coverage density is a feasible way to adjust the wall thickness of the resulting graphene frameworks. It is well-known that the native OA ligands attached to Fe_3_O_4_ NCs can be readily replaced by a different type of ligands through a ligand-exchange process (Neouze and Schubert, 2008). However, the questions as to whether the ligand-exchanged NCs can still self-assemble into 3D ordered superlattices and the subsequent ligand carbonization can still be accomplished without incurring NC sintering remain unresolved.

**Figure 1:**
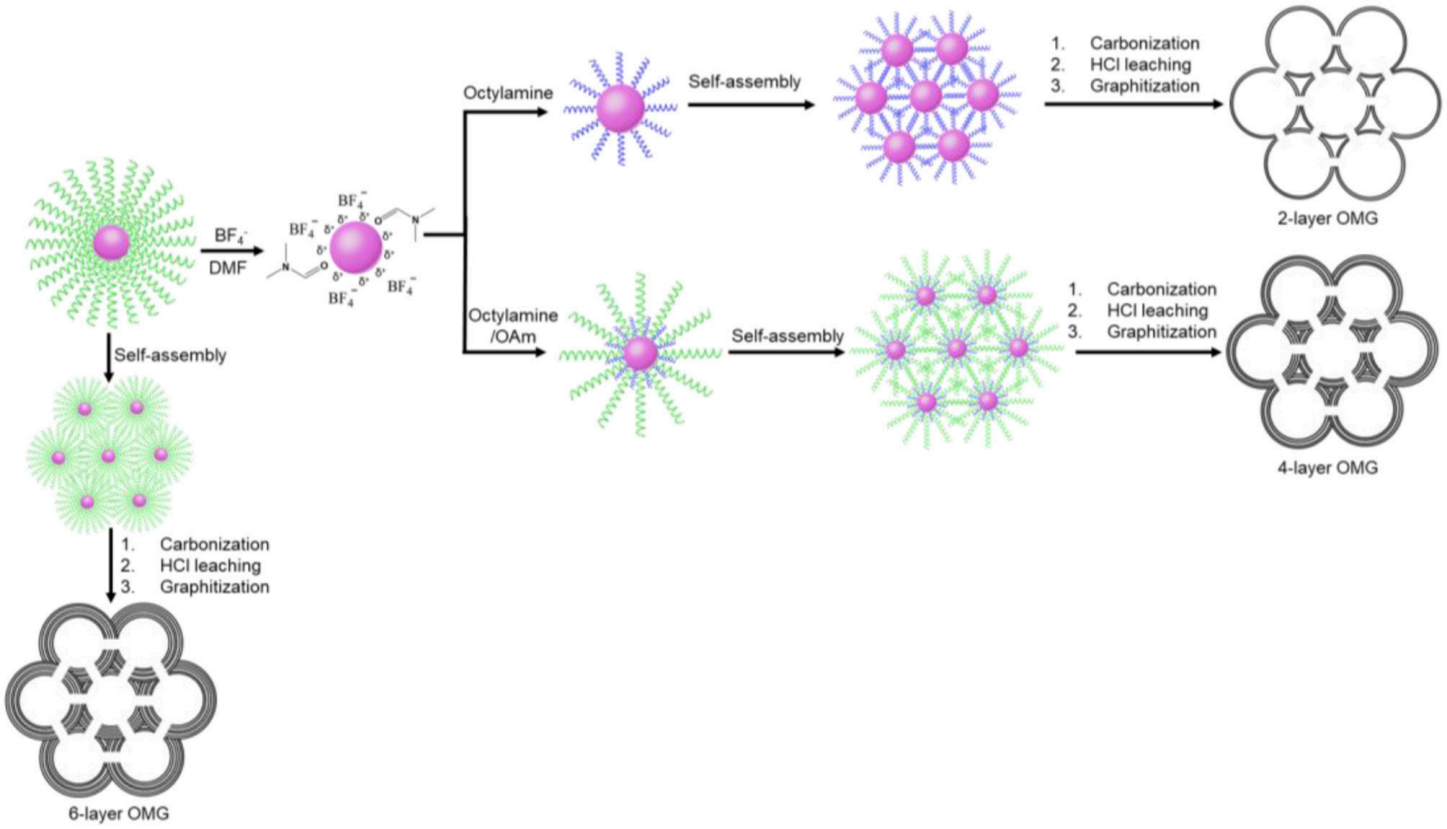
Schematic illustration of the fabrication of the 2-, 4-, and 6-layer OMG.

In this work, a two-step ligand-exchange-reaction strategy is employed to endow Fe_3_O_4_ NCs with different surface-coating hydrocarbon moieties prior to self-assembly. As illustrated in Figure 1, the as-synthesized, OA-capped Fe_3_O_4_ NCs are first treated with HBF_4_, which has been demonstrated to be capable of efficiently stripping off the original OA ligands (Dong et al., 2011). The resulting Fe_3_O_4_ NCs, capped by ${\text{BF}}_{4}^{-}$ anions, can be well redispersed in hydrophilic solvents such as DMF. Another important feature about HBF_4_ treatment is that the ${\text{BF}}_{4}^{-}$-capped Fe_3_O_4_ NCs can act as intermediates, which can be further functionalized by a second ligand of choice (Dong et al., 2011). In order to reduce the wall thickness, a short molecular ligand such as octylamine is used for the sequential ligand exchange, transferring Fe_3_O_4_ NCs back to a hydrophobic solvent such as hexane. The subsequent self-assembly of the octylamine-capped Fe_3_O_4_ NC superlattices followed by ligand carbonization, HCl leaching, and framework graphitization results in OMG typically consisting of 2 layers of graphene (2-layer OMG). The use of both octylamine and oleylamine (OAm) as the co-ligands to treat ${\text{BF}}_{4}^{-}$-capped Fe_3_O_4_ NCs will increase the wall thickness. By tuning the ratio of the two ligands, it is possible to obtain OMG constructed by 3, 4, or 5 layers of graphene (3-, 4-, and 5-layer OMG). Without ligand exchange, the direct conversion of the OA-capped Fe_3_O_4_ NC superlattices typically leads to OMG comprising 6 layers of graphene (6-layer OMG).

**Figure 2:**
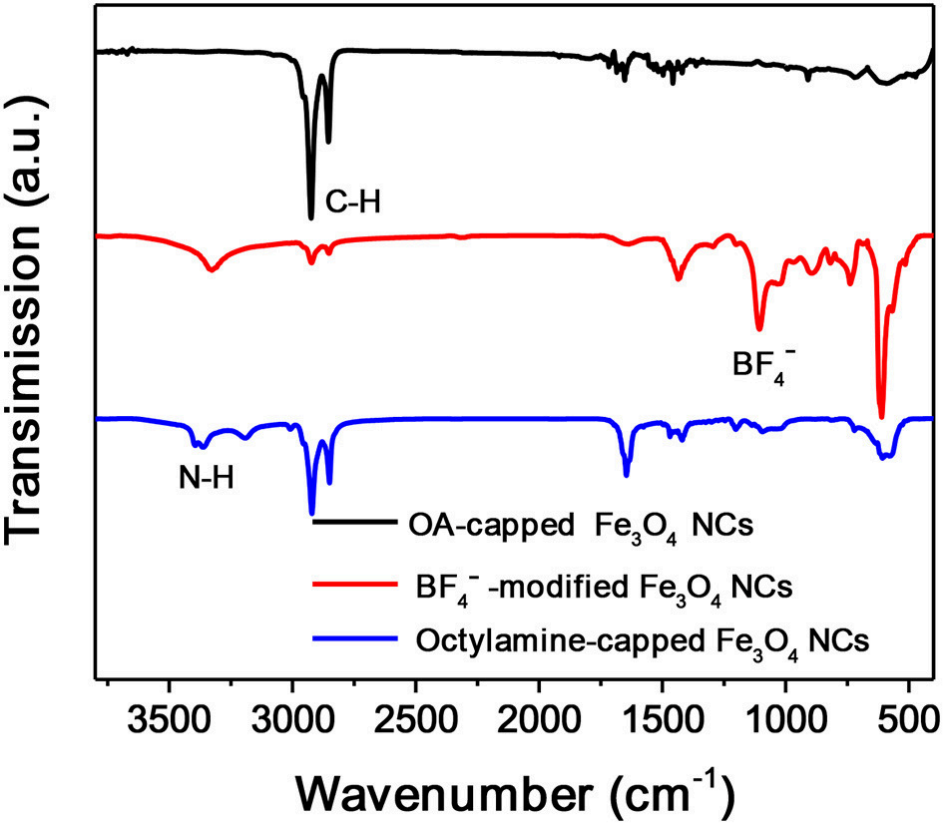
FTIR spectra of Fe_3_O_4_ NCs capped with OA, ${\text{BF}}_{4}^{-}$, and octylamine, respectively.

Octylamine is chosen as a ligand example to demonstrate the formation of OMG. Monodisperse Fe_3_O_4_ NCs with a mean diameter of 14 nm capped by OA are synthesized according to the literature methods (Park et al., 2004). The subsequent ligand-exchange process can be monitored by FTIR spectroscopy. As shown in Figure 2, the intensity of the absorption peaks at 2,800–3,000 cm^−1^, which are the characteristic C-H stretching vibrations of OA molecules, is substantially reduced when the as-synthesized Fe_3_O_4_ NCs are subjected to HBF_4_ treatment. Meanwhile, a new peak around 1,101 cm^−1^ ascribed to ${\text{BF}}_{4}^{-}$ anions emerges, suggesting the nearly complete replacement of OA by ${\text{BF}}_{4}^{-}$ anions, consistent with previous results (Dong et al., 2011). The ${\text{BF}}_{4}^{-}$-capped Fe_3_O_4_ NCs are then treated with octylamine, the accomplishment of which is evidenced by the recovered C-H stretching vibrations at 2,800–3,000 cm^−1^. The emergence of the characteristic N-H stretching vibrations at 3,250–3,500 cm^−1^ and the disappearance of the adsorption peak assigned to ${\text{BF}}_{4}^{-}$ anions also confirm the replacement of ${\text{BF}}_{4}^{-}$ anions by octylamine. Similar to the original Fe_3_O_4_ NCs capped with OA, the octylamine-capped Fe_3_O_4_ NCs can also be dispersed in hexane to form stable colloidal solutions.

### Structural characterization

**Figure 3:**
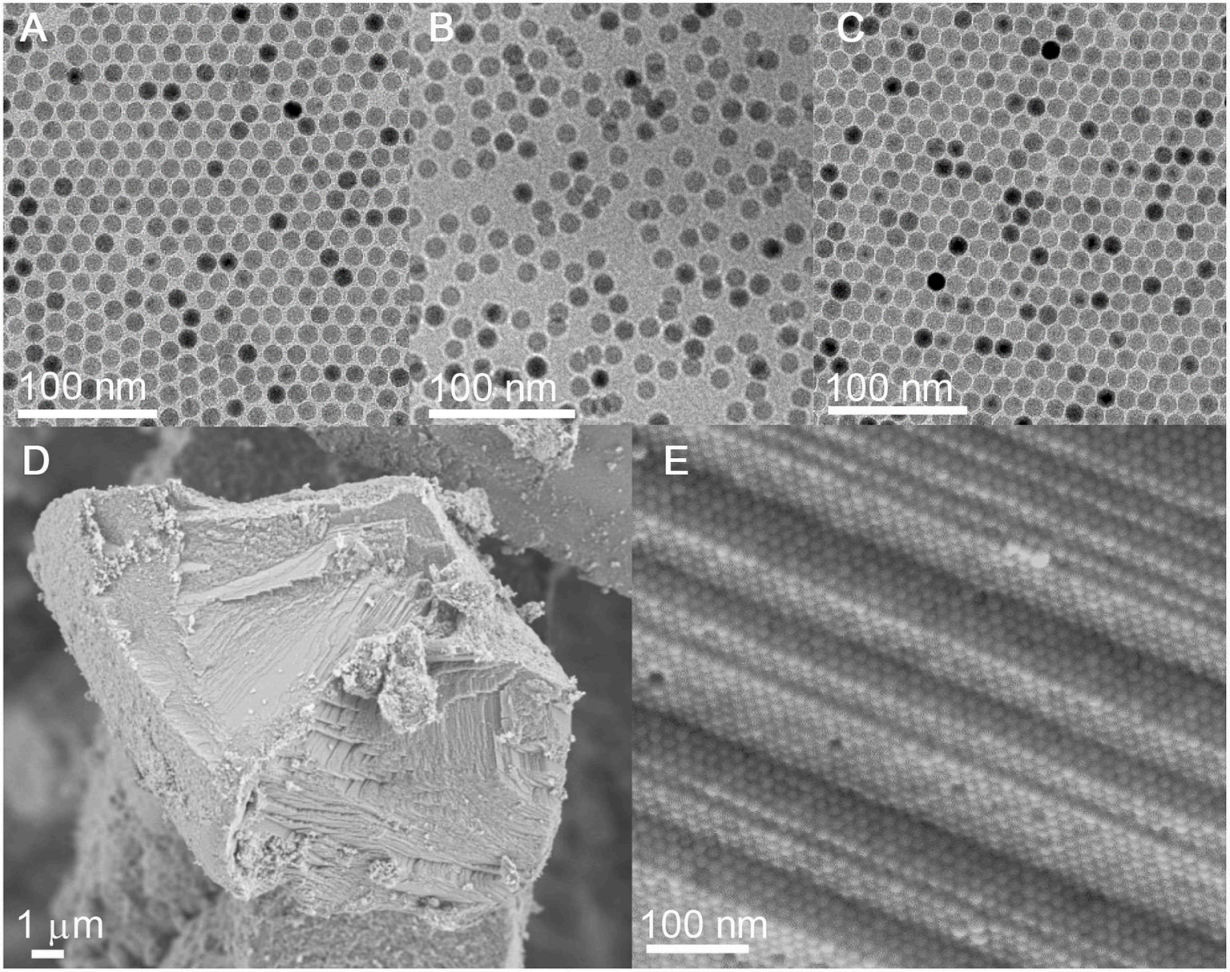
**(A–C)** TEM images of Fe_3_O_4_ NCs capped with OA, ${\text{BF}}_{4}^{-}$, and octylamine, respectively. **(D,E)** SEM and the corresponding HRSEM images of carbonized superlattices derived from the octylamine-capped Fe_3_O_4_ NCs.

TEM shows that the size and the size uniformity of the original Fe_3_O_4_ NCs remain nearly unchanged after the sequential ligand exchange with HBF_4_ and octylamine (Figures 3A–C). Additionally, the octylamine-capped Fe_3_O_4_ NCs can self-assemble into 2D hexagonal arrays on the TEM grid, suggesting that Fe_3_O_4_ NCs are well-capped by octylamine. Besides, the average interparticle spacing between neighboring Fe_3_O_4_ NCs is reduced from ~4.0 to ~1.7 nm, consistent with the shorter chain length of octylamine compared with the initial OA ligands.

The hexane solution containing the octylamine-capped Fe_3_O_4_ NCs is allowed to evaporate under ambient conditions, during which the gradual evaporation of hexane drives the organization of Fe_3_O_4_ NCs. SEM reveals the formation of 3D long-range-ordered NC superlattices after the complete evaporation of hexane. As with the case of the OA-capped Fe_3_O_4_ NCs, the 3D superlattices assembled from the octylamine-capped Fe_3_O_4_ NCs also exhibit a face-centered-cubic (FCC) structure as evidenced by SAXS (Figure S1), suggesting that ligand exchange with octylamine does not alter the self-assembly behavior of Fe_3_O_4_ NCs. Importantly, the superlattice structure is well-retained without NC agglomeration after ligand carbonization performed at 500°C, indicating that octylamine as a ligand is sufficient to inhibit the sintering of NCs during heat treatment (Figures 3D,E and Figure S2). The carbon content in carbon-coated Fe_3_O_4_ NC superlattices is determined to be ~1.69 wt%, much lower than that (~13.57 wt%) of superlattices assembled from the OA-capped Fe_3_O_4_ NCs (**Figure 5A**). This result is reasonable given the much shorter chain length of octylamine relative to OA.

**Figure 4:**
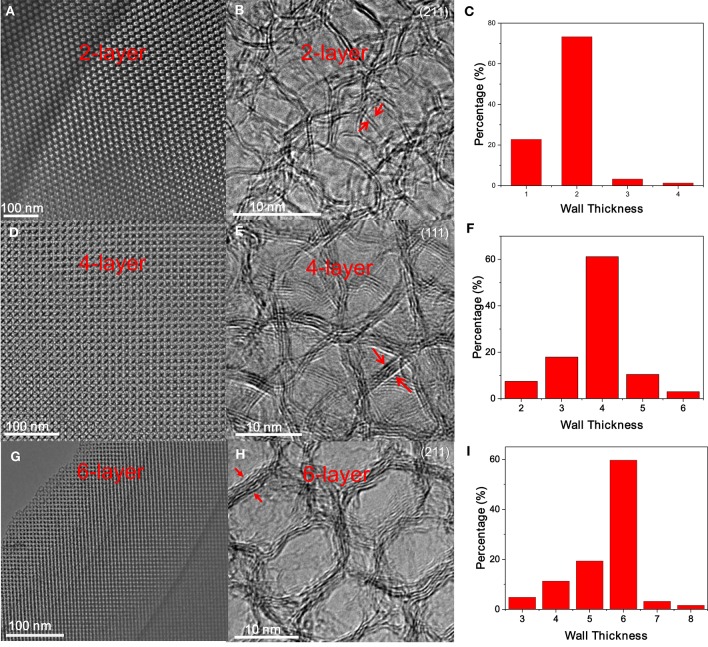
**(A,D,G)** Low-magnification TEM images, **(B,E,H)** the corresponding HRTEM images, and **(C,F,I)** wall thickness distribution of 2-, 4- and 6-layer OMG, respectively.

After ligand carbonization, the carbon-coated Fe_3_O_4_ NC superlattices are treated with HCl, and the complete leaching of Fe_3_O_4_ NCs yields ordered mesoporous frameworks consisting of interconnected carbon shells derived from octylamine. To form OMG, the resulting amorphous carbon frameworks are simply calcined at 1,000°C in Ar. Low-magnification TEM indicates that the resultant OMG possesses a highly ordered mesoporous structure inherited from Fe_3_O_4_ NC superlattices (Figure 4A), while HRTEM reveals that the framework is mainly composed of two layers of graphene (Figure 4B), which is further confirmed by statistical analyses based on HRTEM images (Figure 4C). It is noteworthy that collapsed or disordered frameworks are also observed in some regions when surveying the same OMG sample in TEM (Figure S3), probably due to its ultrathin framework thickness. Nonetheless, our studies establish that tuning the chain length of the surface-coating ligands is indeed a feasible way to adjust the wall thickness while maintaining the structural regularity of OMG. It should also be mentioned that our attempts to further reduce the wall thickness down to a single layer of graphene by using shorter ligands such as butylamine are not successful because severe agglomeration of Fe_3_O_4_ NCs occurs after ligand carbonization (Figure S4), degrading the ordering of NC superlattices. These results indicate that butylamine is not sufficient to inhibit the sintering of Fe_3_O_4_ NCs during ligand carbonization, presumably due to its weaker protective capability arising from the shorter chain length.

**Figure 5:**
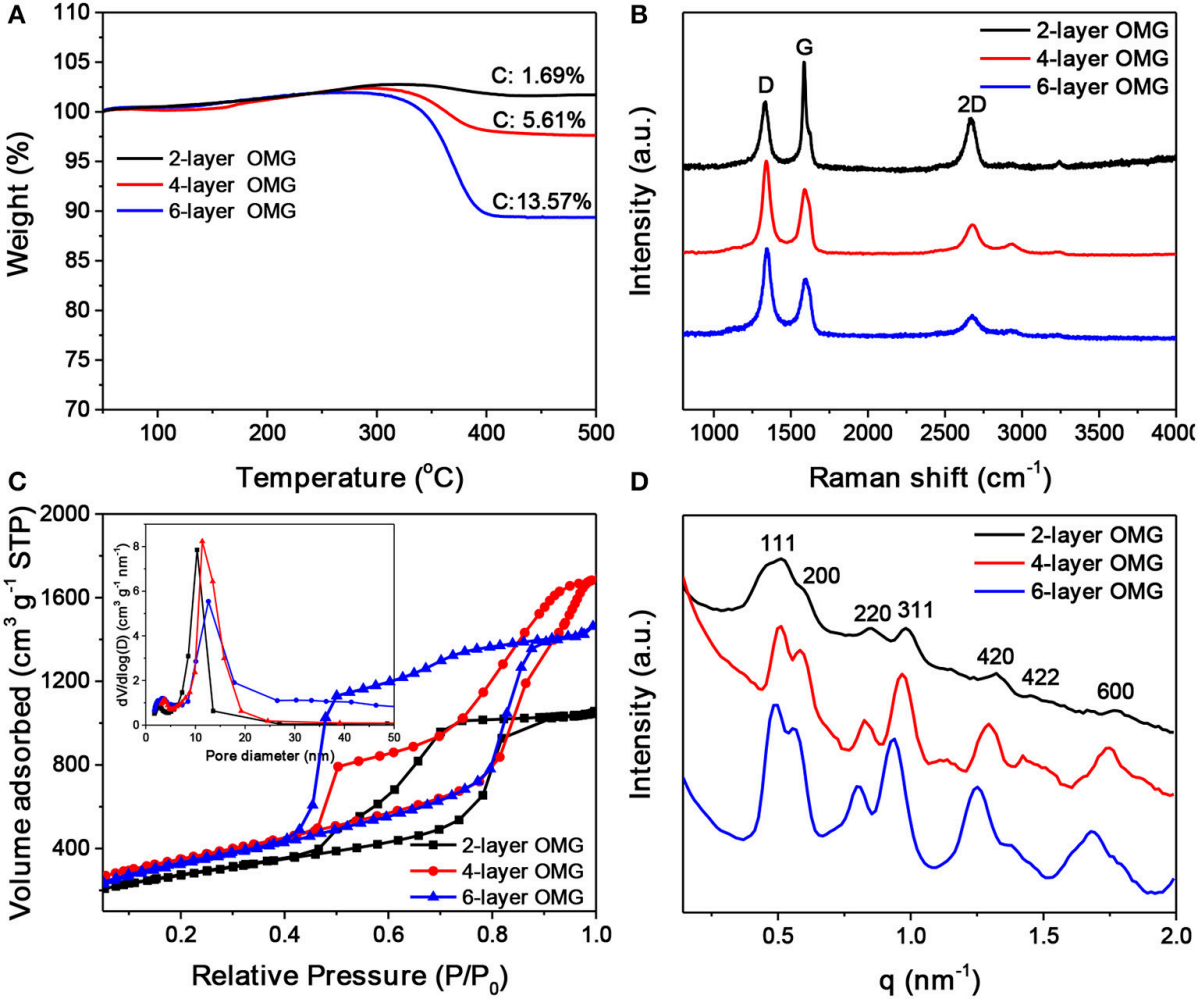
**(A)** TGA scans of carbonized superlattices derived from octylamine-, octylamine/OAm-, and OA-capped Fe_3_O_4_ NCs, respectively. **(B–D)** Raman spectra, N_2_ adsorption-desorption isotherms and pore width distribution (inset), and SAXS patterns of the 2-, 4-, and 6-layer OMG, respectively.

Despite the difficulty to obtain single-layer OMG, we can fine-tune the wall thickness in the range of 2–6 layers by choosing appropriate ligands. For instance, 4-layer OMG is attainable when both octylamine and OAm (2:1 molar ratio) are used as co-ligands to treat the ${\text{BF}}_{4}^{-}$-capped Fe_3_O_4_ NCs, as illustrated in Figure 1. Compared with the octylamine ligand, the capping of octylamine/OAm is expected to increase the average ligand chain length and therefore the carbon content upon ligand carbonization, which is confirmed by TGA. As indicated by TGA (Figure 5A). Calculated from the weight loss of carbon while considering the mass gain arising from the oxidation of Fe_3_O_4_ to Fe_2_O_3_, the carbon content in superlattices derived from the octylamine/OAm-capped Fe_3_O_4_ NCs is about 5.61 wt%, which is higher than that in superlattices consisting of octylamine-capped Fe_3_O_4_ NCs but lower than that in superlattices consisting of OA-capped Fe_3_O_4_ NCs. After acid etching and framework graphitization, TEM (Figure 4D), HRTEM (Figure 4E), and the corresponding statistical analysis (Figure 4F) confirm the formation of ordered mesoporous frameworks constructed mainly by 4 layers of graphene. It is also possible to achieve 3- and 5-OMG by carefully tuning the octylamine and OAm ratio (Figure S5), while direct conversion of superlattices self-assembled from the OA-capped Fe_3_O_4_ NCs without ligand exchange usually yields 6-layer OMG as revealed by TEM, HRTEM and the corresponding statistical analyses (Figures 4G–I).

Raman spectroscopy was carried out to characterize the graphitization degree of various OMG samples. As shown in Figure 5B, all OMG samples possess a high graphitization degree as indicated by the strong *G* and 2*D* bands, consistent with the HRTEM results mentioned above. Further observations indicate that as the wall thickness of OMG is reduced from 6 to 2 layers, the relative intensity of *G* bands (*I*_2*D*_*/I*_*G*_) is gradually increased. Despite the intensity variation, the peak position of the 2*D* bands remains nearly unchanged. This is different from the previous results based on CVD-grown graphene, where the 2*D* band of the few-layer graphene is observed to shift upon the variation of the layer thickness (Ferrari et al., 2006; Gupta et al., 2006). We attribute this discrepancy to the graphene-oxide-like nature of OMG, as the ligand-derived OMG possesses abundant structural defects and oxygen-containing functional groups as indicated by the strong disorder-induced *D* band in Raman spectra (Figure 5B). Besides, the 2-layer OMG exhibits a stronger *G* band compared to the 4- and 6-layer OMG, indicative of a slightly higher degree of graphitization.

Nitrogen adsorption-desorption measurements were performed to analyze the porosity of OMG having different framework thicknesses. As shown in Figure 5C, all the OMG samples exhibit type-IV isotherms with a large H_2_-type hysteresis loop, indicative of the existence of abundant cage-like mesopores. Notably, the desorption pressure is gradually increased from the 6- and 4-layer OMG to the 2-layer OMG. We suspect that the inner gap between two neighboring graphitic carbon layers can offer strong adsorption sites for N_2_, thus the pressure required to desorb N_2_ is decreased with the increased OMG thickness. The specific surface area of 4-layer OMG is ~1,246 m^2^ g^−1^, comparable to that of 6-layer OMG (~1,188 m^2^ g^−1^). Notably, the specific surface area of 2-layer OMG is determined to be ~968 m^2^ g^−1^, much lower than that of both 4- and 6-layer OMG, probably due to the partial structural collapse as mentioned above. For all OMG samples, the pore-width distribution curve demonstrates the presence of two types of mesopores (Figure 5C, inset). The larger pores located at ~10 nm are derived from the leached Fe_3_O_4_ NCs, while the small pores around 3 nm are expected to originate from the interconnected windows and/or the interstitial voids between adjacent large pores. Figure 5D shows the SAXS patterns of the OMG samples with varying wall thicknesses. Both 4- and 6-layer OMG samples exhibit at least seven well-resolved scattering peaks in the SAXS pattern, suggestive of their long-range ordered FCC structure. In comparison, the slightly disordered structural characteristics of the 2-layer OMG, as manifested by the less resolved scattering peaks, is probably caused by the partial framework collapse, in accordance with its lower specific surface area as well. The XRD patterns of 2-, 4-, and 6-layer OMG samples are shown in Figure S6. The two broad peaks centered at 2θ = 24.6° and 43° are assigned to the (002) and (101) planes of stacked graphene layers, respectively. These two peaks become broader and weaker with the decreased thickness of OMG, consistent with a lower degree of periodicity.

### ORR performance

**Figure 6:**
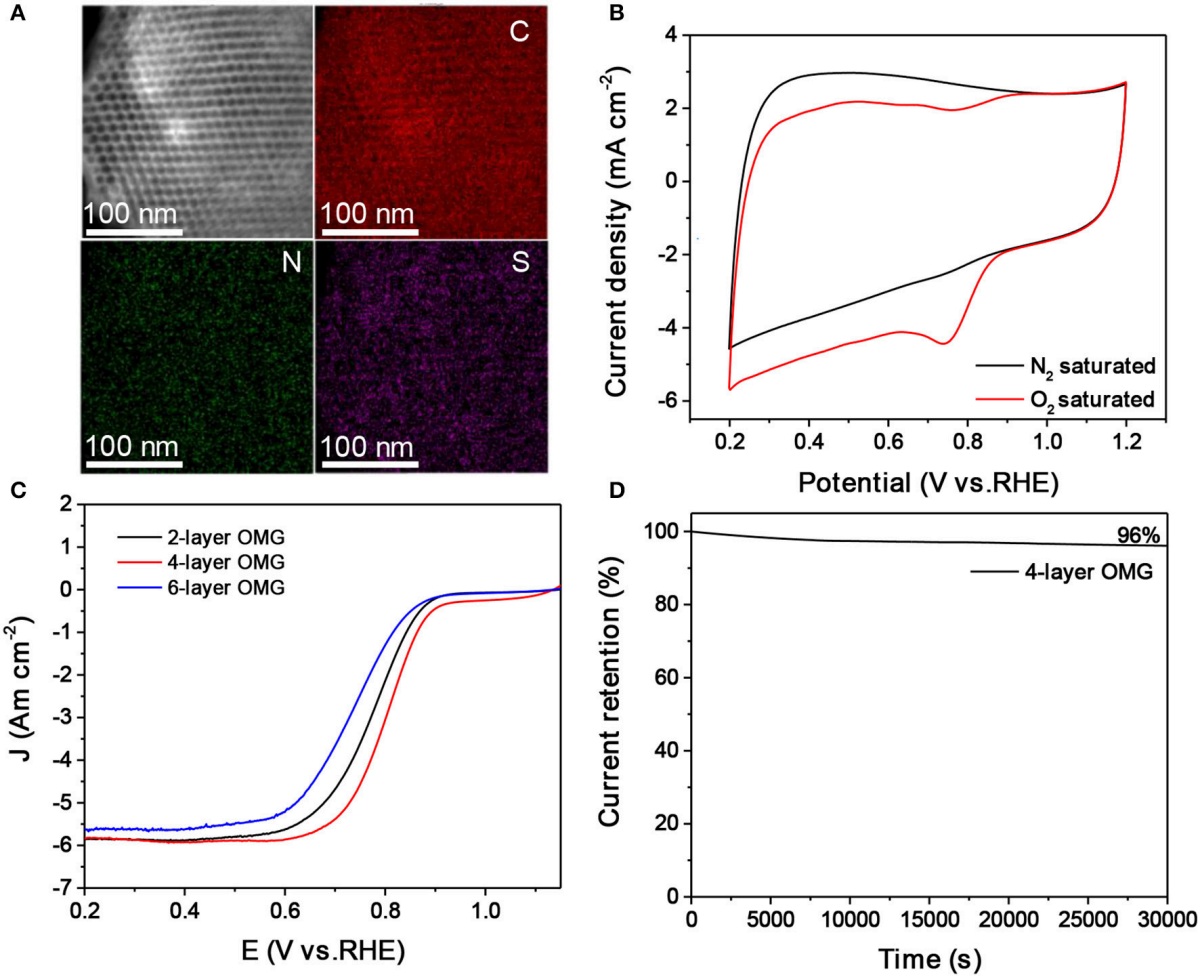
**(A,B)** EDS elemental mapping and CV curves of the N, S-doped 4-layer OMG. **(C)** LSV curves of the 2-, 4-, and 6-layer OMG doped with N and S at a scan rate of 10 mV s^−1^, respectively. **(D)** Time-dependent current density curve of the N, S doped 4-layer OMG.

The surface and interfacial properties of nanomaterials are the key parameters that should be considered in designing high-performance energy materials (Luo et al., 2016, 2017; Yang et al., 2017). The graphene layer thickness is expected to influence the accessibility and utilization of OMG, which are particularly relevant parameters when applying OMG as catalysts. To demonstrate this, OMG with various wall thicknesses are first doped with N and S and are then evaluated as electrocatalysts for ORR. Carbon nanostructures doped with heteroatoms such as N and S have been previously demonstrated to be capable of catalyzing ORR (Zhang et al., 2015). In this work, thiourea is chosen as the dopant precursor to treat all OMG samples by means of pyrolysis. STEM and the corresponding elemental mapping confirm the homogenous doping of N and S within graphene frameworks (Figure 6A). Despite the different wall thicknesses, a comparable amount of N and S has been incorporated into the graphene framework with a doping level of ~3.0–4.0 at% and ~1.0–1.5 at%, respectively, as indicated by EDS analyses. Figure 6B presents the CV curves of N, S-doped 4-layer OMG, collected in 0.1 M KOH saturated with N_2_ and O_2_ respectively. When the electrolyte is saturated with N_2_, the N, S-doped 4-layer OMG displays a featureless CV curve, while a well-defined cathodic peak at ~0.74 V is observed when O_2_ is introduced, suggesting its effective ORR activity in the alkaline medium. To demonstrate the difference of framework thickness on ORR activity, the rotating disk electrode (RDE) technique is employed at a scanning rate of 10 mV s^−1^. Figure 6C shows the polarization curves obtained at 1,600 rpm. Significantly, the N, S-doped 4-layer OMG displays the best catalytic performance among various OMG samples with the half-wave potential at 0.80 V, higher than that of their 2- and 6-layer counterparts. As the 4- and 6-layer OMG samples possess comparable surface areas and doping levels, the higher ORR activity of the 4-layer OMG relative to its 6-layer counterpart could be attributed to its thinner wall thickness, which is favorable for exposing more active sites as well as for facilitating mass and electron transport. Notably, the inferior ORR activity of the 2-layer OMG relative to the 4-layer OMG is probably attributed to its lower surface area caused by the partial framework collapse. Even though, the 2-layer OMG still exhibits a better ORR activity than the 6-layer OMG despite its lower surface area, further demonstrating that the wall thickness indeed has a significant impact on the catalytic activity. In addition to the superior ORR activity, the N, S-doped 4-layer OMG displays 96% current retention after 30,000 s of operation, demonstrating its outstanding electrochemical durability (Figure 6D). In contrast, the 4-layer OMG without N, S doping exhibits the worst ORR performance (Figure S7), further illustrating the necessity of doping to boost the ORR activity of OMG.

## Conclusions

In conclusion, our studies have established that engineering the length of the surface-coating hydrocarbon ligands is a feasible and efficient way to fine-tune the wall thickness of mesoporous graphene. By exploiting the ligand-exchange capability of colloidal NCs, we are able to rationally adjust the wall thickness from 2 to 6 layers of graphene without compromising the highly ordered mesoporous structure of OMG. When evaluated as the electrocatalysts for ORR, the 4-layer OMG doped with S and N exhibits better catalytic performance than its 2- and 6-layer counterparts. The enhanced ORR activity of the 4-layer OMG is attributed to the enhanced exposure of activity sites arising from the ultrathin wall thickness and high surface area.

## Author contributions

DH and AD conceived and designed the experiments. DH performed the experiments carried out data analyses. BW, YY, and SX helped with TEM image capture. TL, GG, and DY helped with ORR measurements. DH and AD wrote the manuscript. All authors discussed the results and commented on the manuscript.

### Conflict of interest statement

The authors declare that the research was conducted in the absence of any commercial or financial relationships that could be construed as a potential conflict of interest.
