# Supplementary material for: Fine-Tuning the Wall Thickness of Ordered Mesoporous Graphene by Exploiting Ligand Exchange of Colloidal Nanocrystals
Source: Front Chem. 2017 Dec 13;5:117. doi: 10.3389/fchem.2017.00117 (PMC5733481; doi:10.3389/fchem.2017.00117)
Supplement: Supplementary file 1 [file DataSheet1.PDF]

Supplementary Material

Fine-tuning the Wall Thickness of Ordered Mesoporous Graphene by Exploiting Ligand Exchange of Colloidal Nanocrystals

Dandan Han[1], Yancui Yan[2], Jishi Wei[1], Biwei Wang[1], Tongtao Li[1], Guannan Guo[2], Dong Yang[2], Songhai Xie[1]*, Angang Dong[1]*
Collaborative Innovation Center of Chemistry for Energy Materials, Shanghai Key Laboratory of Molecular Catalysis and Innovative Materials, Department of Chemistry, Fudan University, Shanghai, China, 2 Department of Macromolecular Science, Fudan University, Shanghai, China

* Correspondence: agdong@fudan.edu.cn (A.D.); shxie@fudan.edu.cn (S.X.)

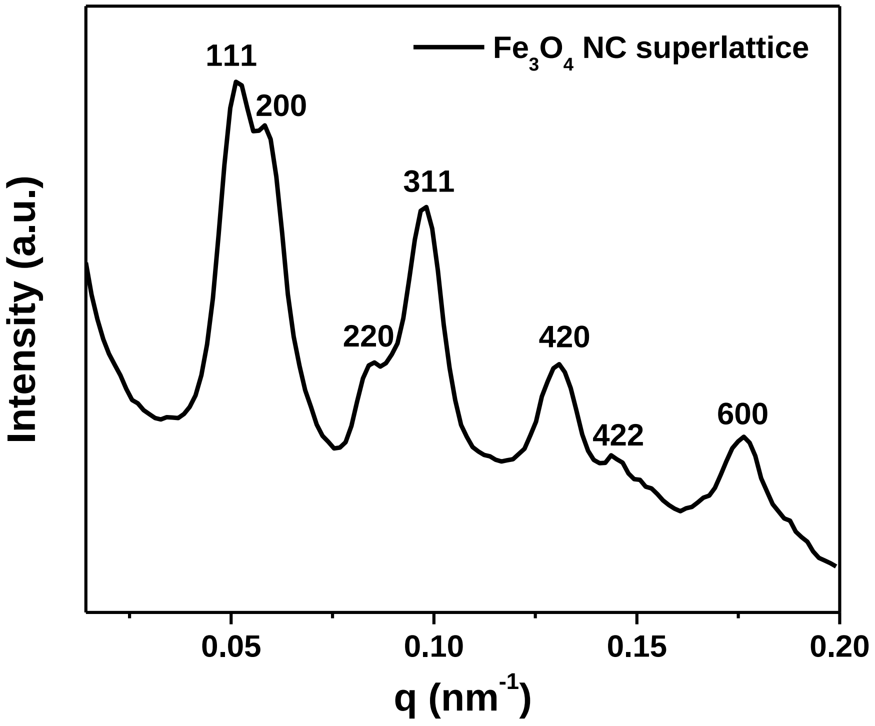

FIGURE S1 SAXS pattern of superlattices as-assembled from the octylamine-capped Fe_3O_4 NCs.

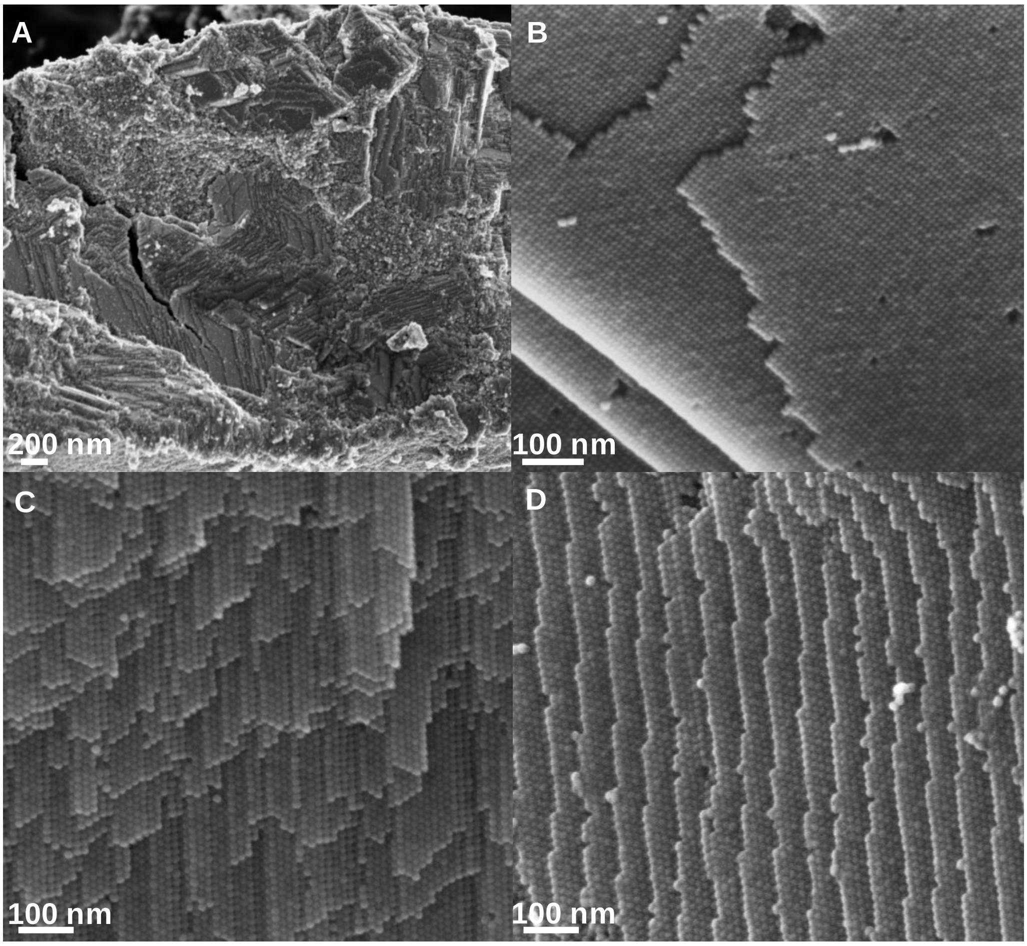

FIGURE S2 (**A**) Low-magnification SEM image and (**B, C, D**) the corresponding HRSEM images of carbonized Fe_3O_4 NC superlattices derived from the octylamine-capped NCs.

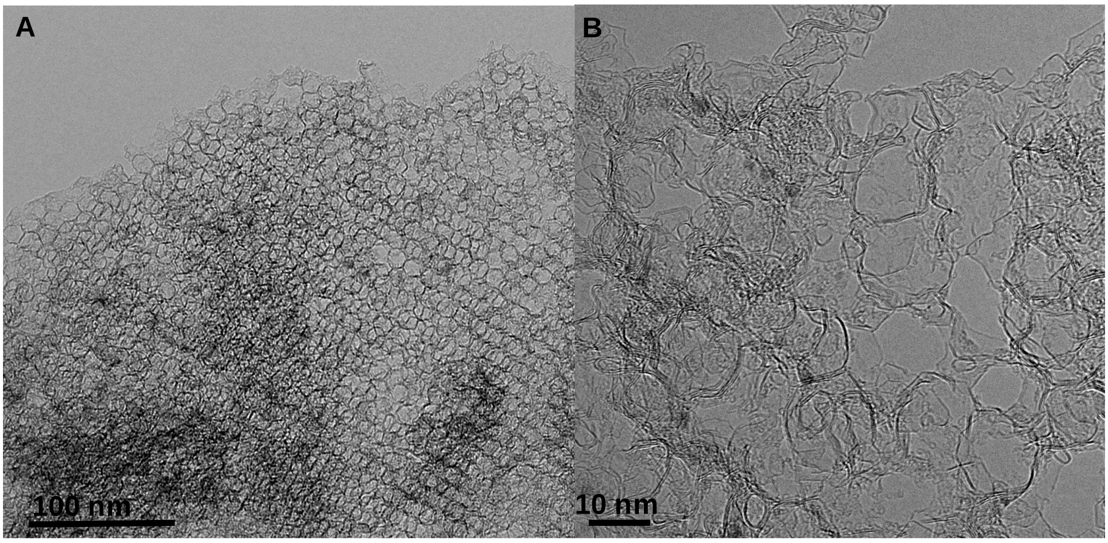

FIGURE S3 (A, B) TEM image and the corresponding HRTEM image of partially collapsed graphene frameworks in the 2-layer OMG.

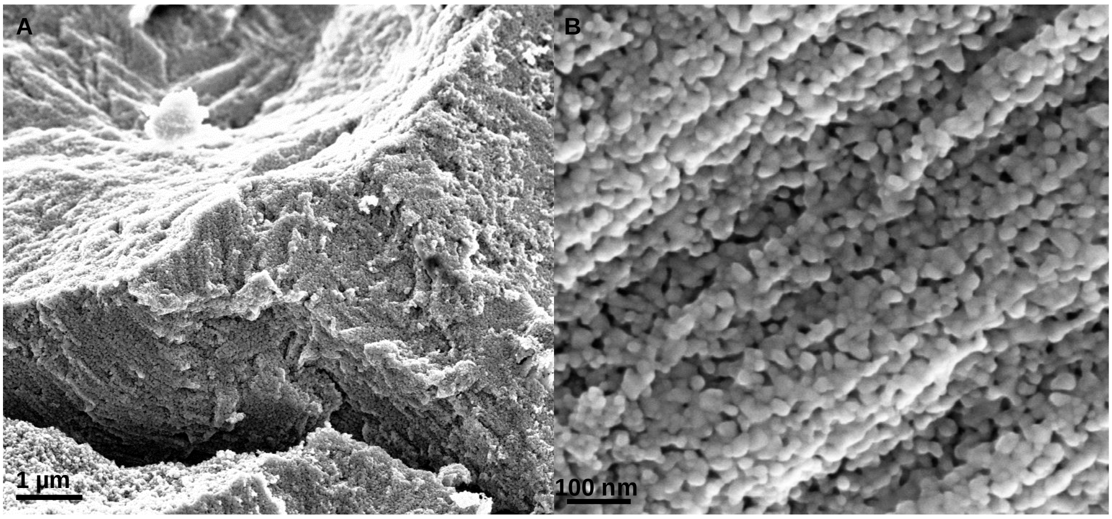

FIGURE S4 (A, B) SEM and the corresponding HRSEM of carbonized superlattices consisting of butylamine-capped Fe_3O_4 NCs.

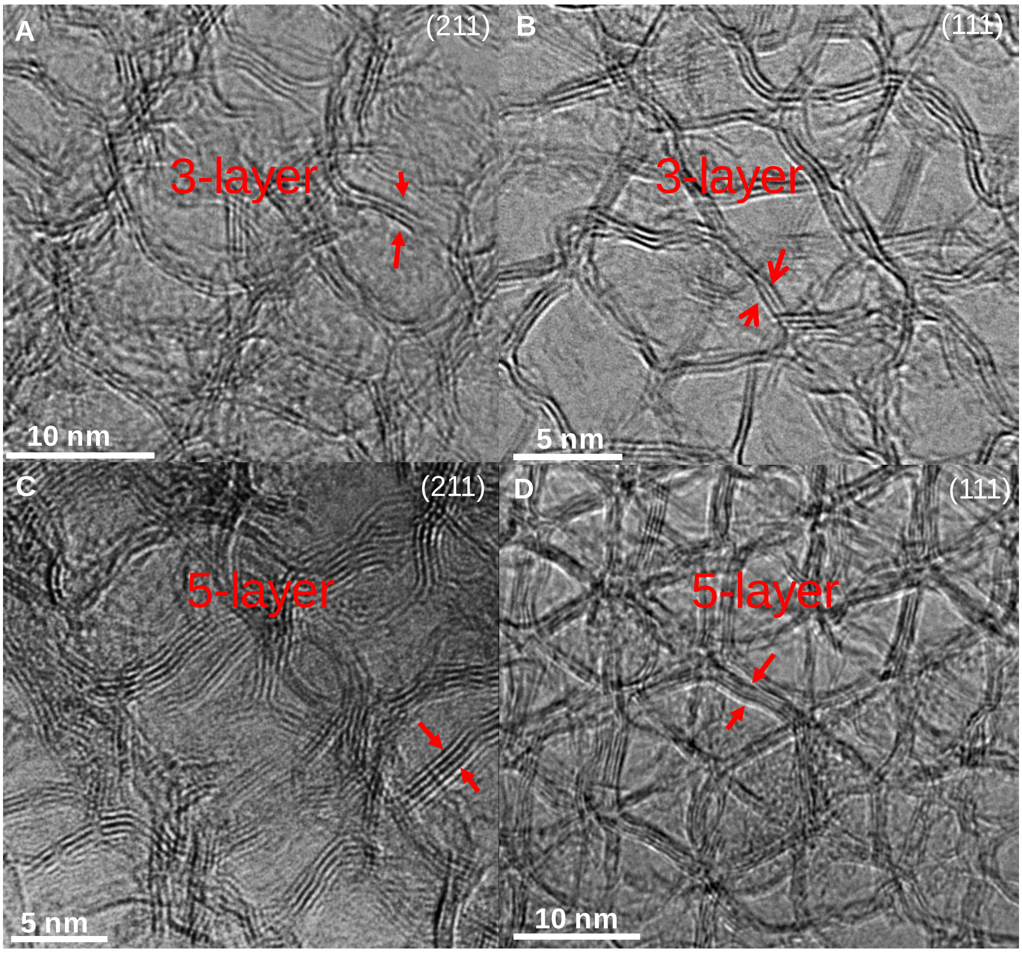

FIGURE S5 HRTEM images of 3- (**A, B**) and 5-layer OMG (**C, D**) along the (211) and (111) directions, respectively.

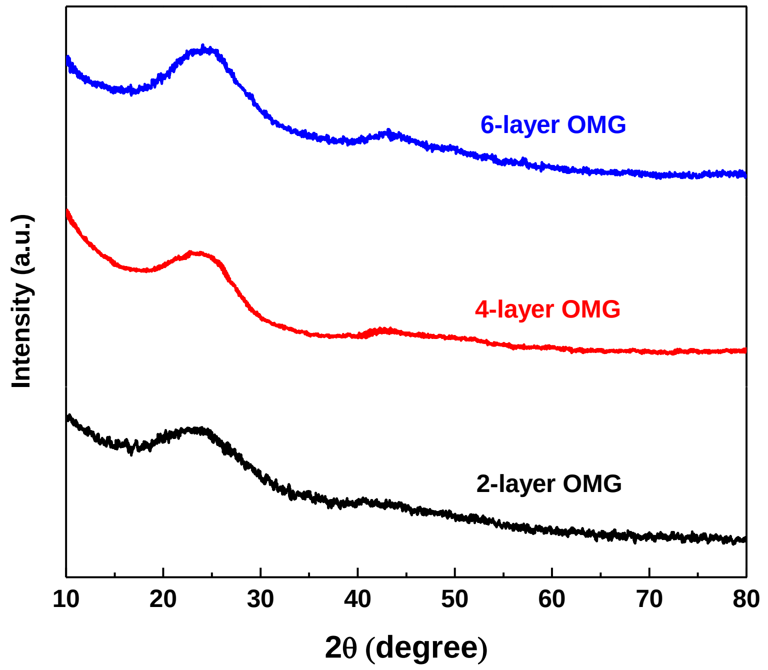

FIGURE S6 XRD patterns of the 2-, 4- and 6-layer OMG samples, respectively.

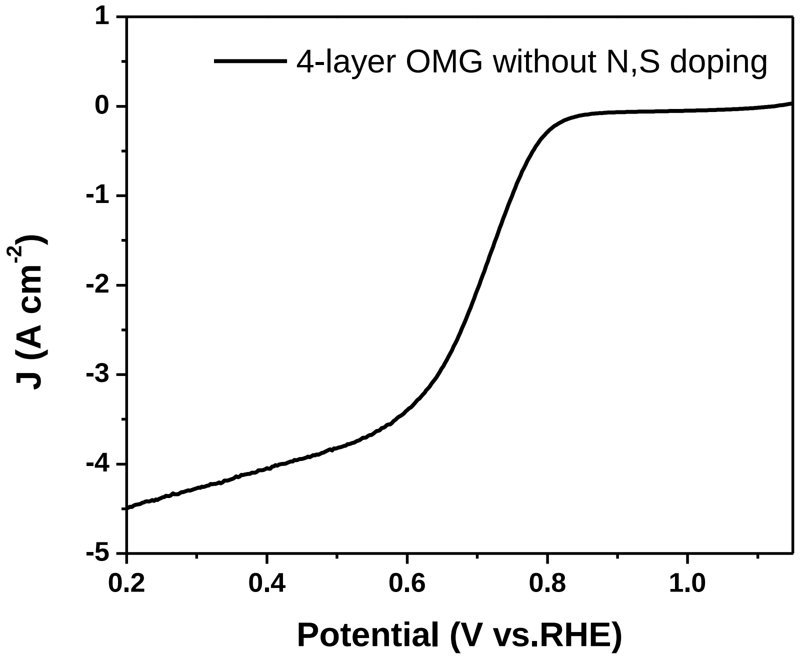

FIGURE S7 LSV curve of the 4-layer OMG sample without N, S doping at a scan rate of 10 mV s^{-1}.
